# Ultrasound‐trigged micro/nanorobots for biomedical applications

**DOI:** 10.1002/SMMD.20230003

**Published:** 2023-04-11

**Authors:** Danqing Huang, Lijun Cai, Ning Li, Yuanjin Zhao

**Affiliations:** ^1^ Institute of Translational Medicine Nanjing Drum Tower Hospital Medical School Nanjing University Nanjing China; ^2^ State Key Laboratory of Bioelectronics School of Biological Science and Medical Engineering Southeast University Nanjing China

**Keywords:** asymmetric acoustic streaming, bubble oscillation, robots, ultrasound

## Abstract

Micro‐ and nanorobots (MNRs) propelled by external actuations have broad potential in biomedical applications. Among the numerous external excitations, ultrasound (US) features outstanding practical significance with merits of its noninvasiveness, tunability, penetrability, and biocompatibility. Attributing to various physiochemical effects of US, it can propel the MNRs with sophisticated structures through asymmetric acoustic streaming, bubble oscillation, and so on. In this review, we introduce several advanced and representative US‐propelled MNRs with inhomogeneous density distribution, asymmetric shape, hollow cavity, etc. The potential biomedical applications of these cutting‐edge MNRs are also presented, including intracellular delivery, harmful substances collection, and so on. Furthermore, we conclude the advantages and limitations of US‐propelled MNRs and prospect their future developments in multidisciplinary fields.

## Graphical abstract

Ultrasound (US)‐propelled micro‐ and nanorobots (MNRs) have shown their bright future in multidisciplinary fields. In this review, several advanced and representative US‐propelled MNRs with inhomogeneous density distribution, asymmetric shape, hollow cavity, and other features are presented. The potential biomedical applications of these cutting‐edge MNRs are also introduced, including intracellular delivery, harmful substances collection, etc. Finally, the advantages and limitations of US‐propelled MNRs are concluded, and their future developments in multidisciplinary fields are also prospected.

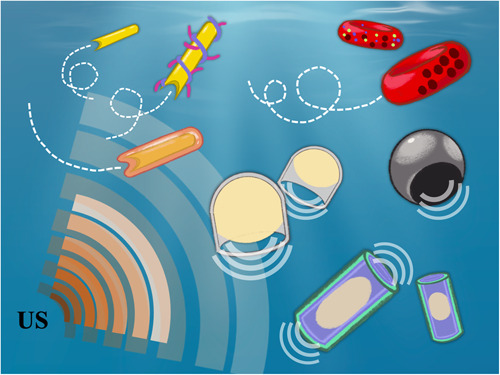

1

**Key points**

- We review the recent progress of ultrasound‐propelled micro‐ and nanorobots and their biomedical applications.
- We focus on the micro‐ and nanorobots with asymmetric geometry and hollow cavity.
- We prospect the potential applications of the micro‐ and nanorobots in multidisciplinary fields.

## 2 INTRODUCTION

Autonomous robots in micron and nano scales hold a bright future in multidisciplinary fields.^1^, ^2^, ^3^, ^4^, ^5^, ^6^, ^7^, ^8^, ^9^, ^10^, ^11^, ^12^, ^13^, ^14^ Attributing to the propulsion provided by fuel‐catalysis or external excitations, the micro/nanorobots (MNRs) display dynamic locomotion in fluid environments.^1^, ^5^, ^6^, ^7^, ^8^, ^9^, ^10^, ^11^, ^15^, ^16^ Compared with the Brownian motion of passive micro/nanoparticles, the controllable actuation of MNRs allows them to proceed at high speed to target locations and to overcome the viscous drag caused by low Reynolds number in complex biological environments.^17^ The sophisticated structures and bio‐modifiable surfaces along with the on‐demand locomotion impart these MNRs with remarkable potentials in biomedical applications, including chemical detections, biodetoxification, biosensing, drug delivery, microsurgery, and so on. MNRs propelled by chemical catalysis have inevitable defects due to the relative toxic fuel and finite chemical reactions.^1^, ^6^, ^7^, ^8^ By contrast, MNRs propelled by external stimulation, such as light, electric, magnetic, and sound field, can effectively address the limitations of chemical fuels.^18^, ^19^, ^20^, ^21^, ^22^, ^23^, ^24^, ^25^, ^26^, ^27^, ^28^ Especially, the on‐demand manipulation of external fields allows remote and real‐time control of the MNRs located deep in the tissue. However, the biocompatibility of these external power fields is most decisive for their biomedical applications.

As a mechanical wave that has been widely applied in medical diagnosis and treatment for a long time, ultrasound (US) features both certain biological safety and deep tissue accessibility within a certain frequency and power range.^13^, ^29^, ^30^, ^31^, ^32^, ^33^, ^34^, ^35^, ^36^, ^37^, ^38^, ^39^ Unlike other external power sources using electric and magnetic fields that are based on bulky and expensive equipment to realize strong penetration and large field coverage, US with tunable frequencies and powers can be generated through piezoelectric components. Additionally, the US itself shows extremely high energy density and low attenuation when propagating through fluid environments, paving the way for its potential application in propelling MNRs sited deeply in the tissue.^13^, ^14^, ^17^, ^40^, ^41^, ^42^, ^43^, ^44^, ^45^, ^46^, ^47^, ^48^, ^49^, ^50^, ^51^, ^52^ US displays numerous chemical and physical effects due to its periodic mechanical vibration when interacting with the medium. Cavitation effect, the best known of these physiochemical effects, involves the generation, oscillation, expansion, and collapse of microbubbles in liquid, which are accompanied by the transformation and release of acoustic energy.^30^, ^31^, ^53^, ^54^, ^55^, ^56^, ^57^, ^58^, ^59^, ^60^ In contrast, the mechanical vibration of US also causes the microstreaming of fluids, providing propulsion for the locomotion of substances in liquids.^61^, ^62^, ^63^ Combining with many other physiochemical phenomena, US serves as an unprecedented candidate for the actuation of MNRs in biological environments.

In recent decades, US‐propelled MNRs with distinctive structures and multifaceted functions have been widely explored.^12^, ^13^, ^14^, ^17^, ^40^, ^41^, ^42^, ^43^, ^44^, ^45^, ^46^, ^47^, ^48^, ^49^, ^50^, ^51^, ^52^ The locomotion of the MNRs also developed from marching back and forth in a one‐dimensional plane, moving in multiple directions in a two‐dimensional (2D) plane, to rising and falling at controllable altitude in three‐dimensional (3D) space. In addition to the on‐demand control of locomotion velocity and navigation, by modifying the surface with bioactive substances or encapsulating therapeutic agents inside the MNRs, various biomedical applications can be achieved, like targeted drug delivery and release, toxicant detection and collection, and nano‐engineering in cellular level and hard‐to‐reach spaces, etc. In this review, we introduced some representative and advanced US‐propelled MNRs and their biomedical applications, including those based on asymmetric acoustic streaming actuation, bubble oscillation propulsion, and so on (Table 1). We also concluded the advantages and limitations of the US‐propelled MNRs and prospected their potential applications in multidisciplinary fields.

**TABLE 1:** The presented US‐propelled MNRs and corresponding US parameters.

Propelled mechanism	MNRs	US parameters	Ref.
Asymmetric acoustic streaming actuation	siRNA‐modified AuNWs	6.0 V, 2.66 MHz	44
	Dual‐cell membrane‐coated AuNWs	2.0 V, 2.66 MHz	50
	Pt micromotor	3.0 V, 3.24 MHz	48
	RBC micromotor	3.0 V, 2.93 MHz	46
	Multiple cargo loaded RBC micromotor	4.0 V, 2.40 MHz	47
Bubble oscillation propulsion	Multi‐scale cup‐shaped swimmers (3 μm)	0.05–0.15 V, 1.18–1.45 MHz	41
	Multi‐scale cup‐shaped swimmers (1 μm)	0.4 V, 4.89 MHz	41
	Multi‐scale cup‐shaped swimmers (600 nm)	2.0 V, 8.01–11.21 HMz	41
	Multi‐scale cup‐shaped swimmers (500 nm)	1.5 V, 17.91 MHz	41
	3D‐printed microrobots	2–4 V, 350–480 kHz	43
	Tubular hydrophobic microrobots	15 V, 4.6 MHz	40
	3D swimming microdrone	22–29 V, 5.9–11.7 kHz	42

Abbreviations: 3D, three‐dimensional; AuNWs, gold nanowires; Pt, platinum; RBC, red blood cell; US, ultrasound.

## 3 ASYMMETRIC ACOUSTIC STREAMING ACTUATION

**FIGURE 1:**
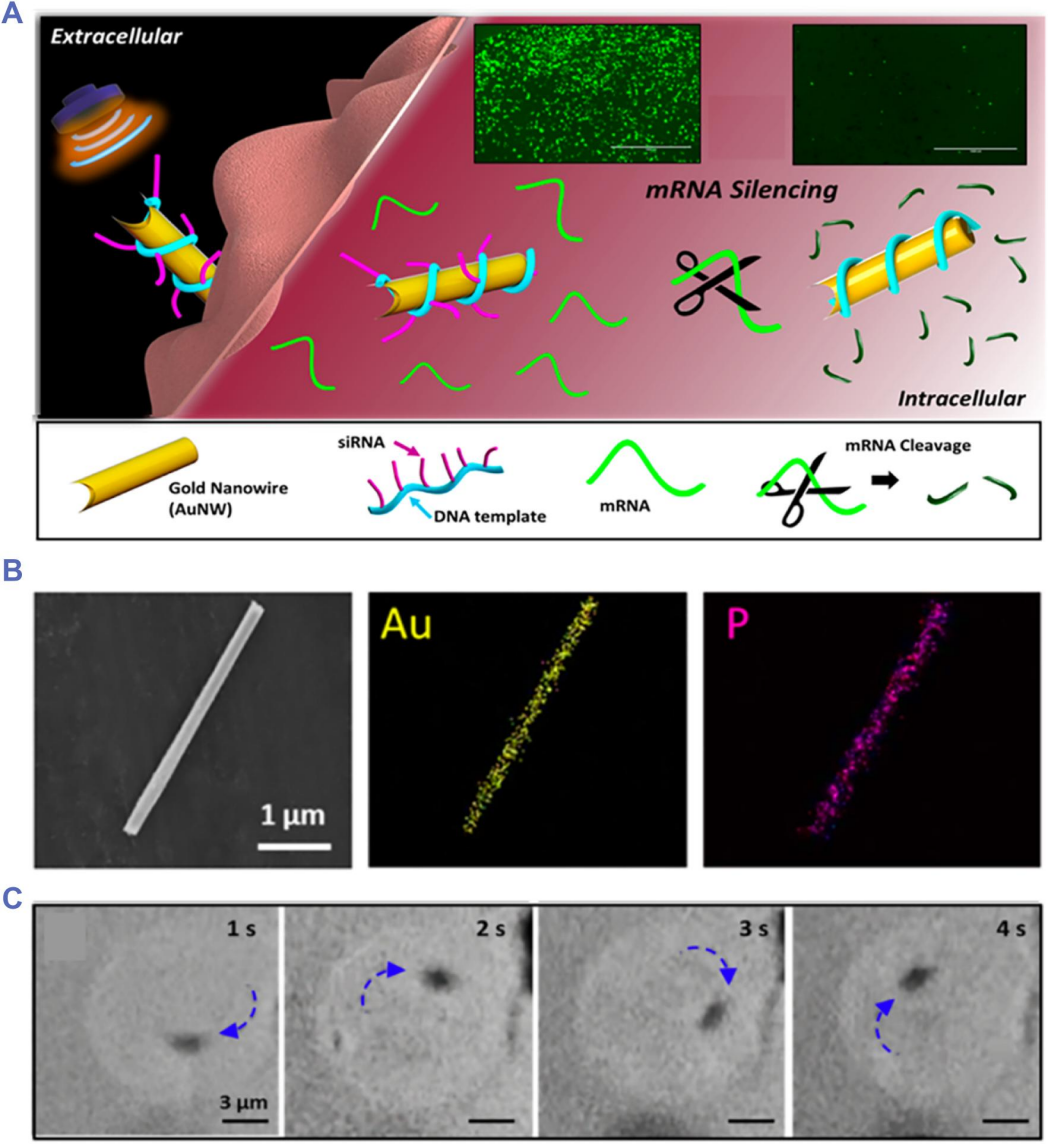
Ultrasound (US)‐propelled gold nanowires (AuNWs) for intracellular gene delivery. (A) Schematic illustration of the siRNA‐modified AuNWs for US‐actuated intracellular gene delivery. (B) Scanning electron microscope (SEM) images of the AuNW. (C) Real‐time images of the US‐propelled spinning motion of an AuNW inside a cell. *Source*: Reproduced with permission.^44^ Copyright 2016, American Chemical Society.

In metallic micro‐ or nanoparticles with asymmetric shape, taking gold nanowire (AuNW) for instance, the two ends of which are convex and concave,^64^, ^65^, ^66^, ^67^ US can steer AuNW with strong axial directional actuation toward the convex end. It is because, under the US excitation, the concave end concentrates the acoustic energy while the convex end weakens the energy. In brief, the asymmetric shape of the AuNW brings about the asymmetric distribution of acoustic energy, rendering the end with more acoustic pressure to push the end with lower pressure. Therefore, based on the sensitive US‐propelling activity of AuNWs, Ávila et al. applied modified AuNWs as efficient siRNA carriers for intracellular gene delivery (Figure 1A,B).^44^ The dynamic locomotion of US‐propelled AuNWs can easily breakthrough biological barriers to achieve cargo delivery, compensating the limitations during commonly applied gene delivery (Figure 1C). As far as the US sensitivity and kinetic performance are concerned, AuNWs are still foreign substances for organisms and will cause inevitable rejection reactions.

**FIGURE 2:**
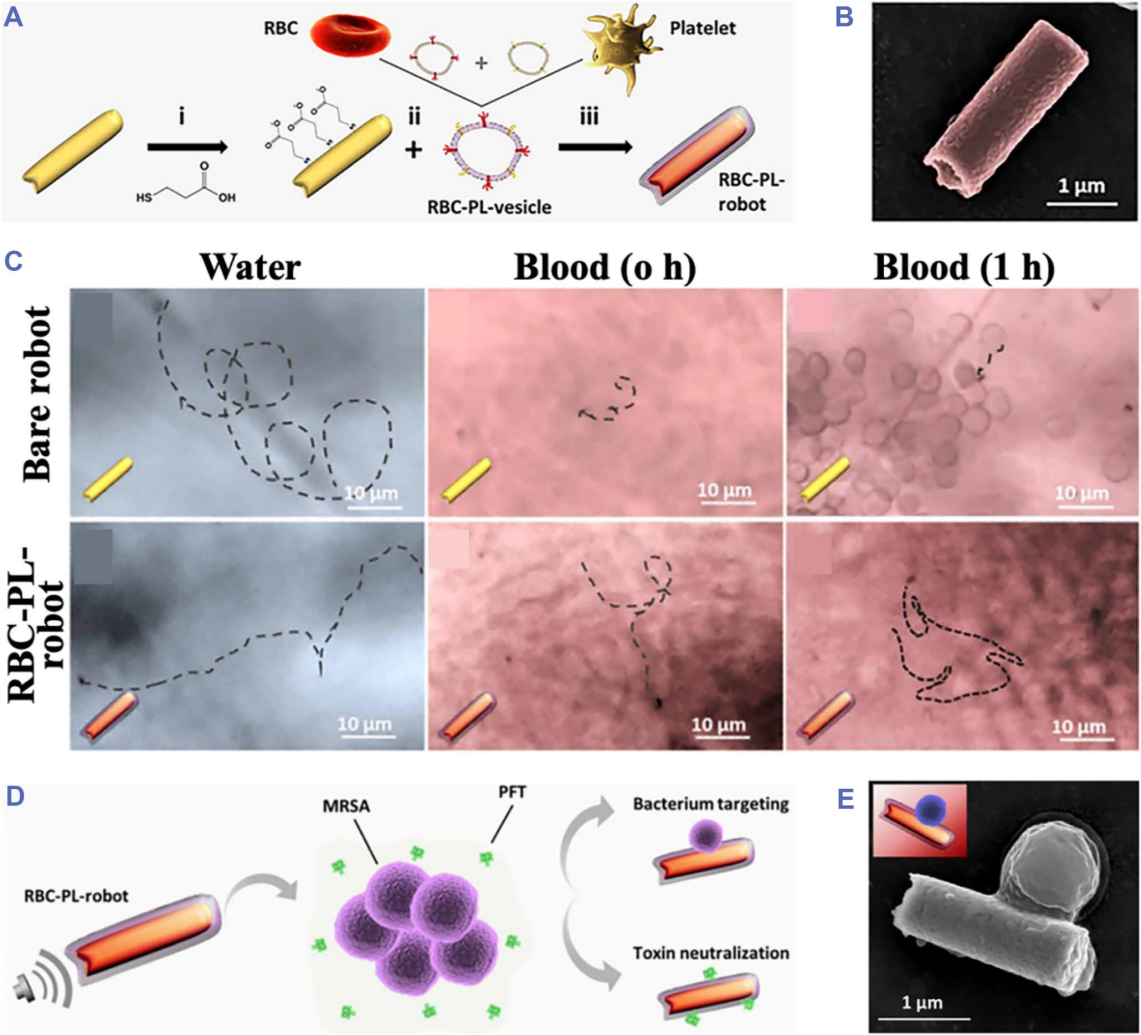
Dual‐membrane encapsulated gold nanowires (AuNWs) for pathogen collection. (A) Schematic illustration of the dual membrane‐coated AuNW. (B) Scanning electron microscope (SEM) image of the RBC‐PL‐robot. (C) The locomotion of bare and red blood cells (RBC) and platinum (PT) membranes coated the robot in water and whole blood. (D) Schematic illustration of the RBC‐PL‐robot for pathogen targeting and detoxification. (E) SEM image of an RBC‐PL‐robot capturing a bacterium. *Source*: Reproduced with permission.^50^ Copyright 2018, The Authors, published by American Association for the Advancement of Science.

Therefore, to address the limited biocompatibility of AuNWs and improve their accessibility toward target cells, Ávila's group went one step further and proposed the application of biogenic materials to coat the AuNWs (Figure 2A).^50^ Cell membrane coating has been elucidated to endow inorganic materials with biological functions and biocompatibility.^68^, ^69^, ^70^, ^71^, ^72^, ^73^ By coating the AuNWs with membranes from red blood cells (RBCs) and platelets (PTs), Ávila and co‐workers fabricated dual–cell membrane–functionalized nanorobots (RBC‐PL‐robots) (Figure 2B). In water, both the bare and membrane‐coated AuNWs showed efficient locomotion. However, in whole blood, compared with bare AuNW robots, the RBC‐PL‐robots displayed faster movements before and after 1 h incubation (Figure 2C). Moreover, ascribing to the bio‐functional proteins of RBC and platelet membranes, the RBC‐PL‐robots showed excellent performance in targeting pathogens and neutralizing toxins (Figure 2C,D). The unprecedented explorations of Ávila's group displayed the multifaceted functions of Au nanomaterials, shedding light on the biomedical applications of US‐propelled nanorobots.

**FIGURE 3:**
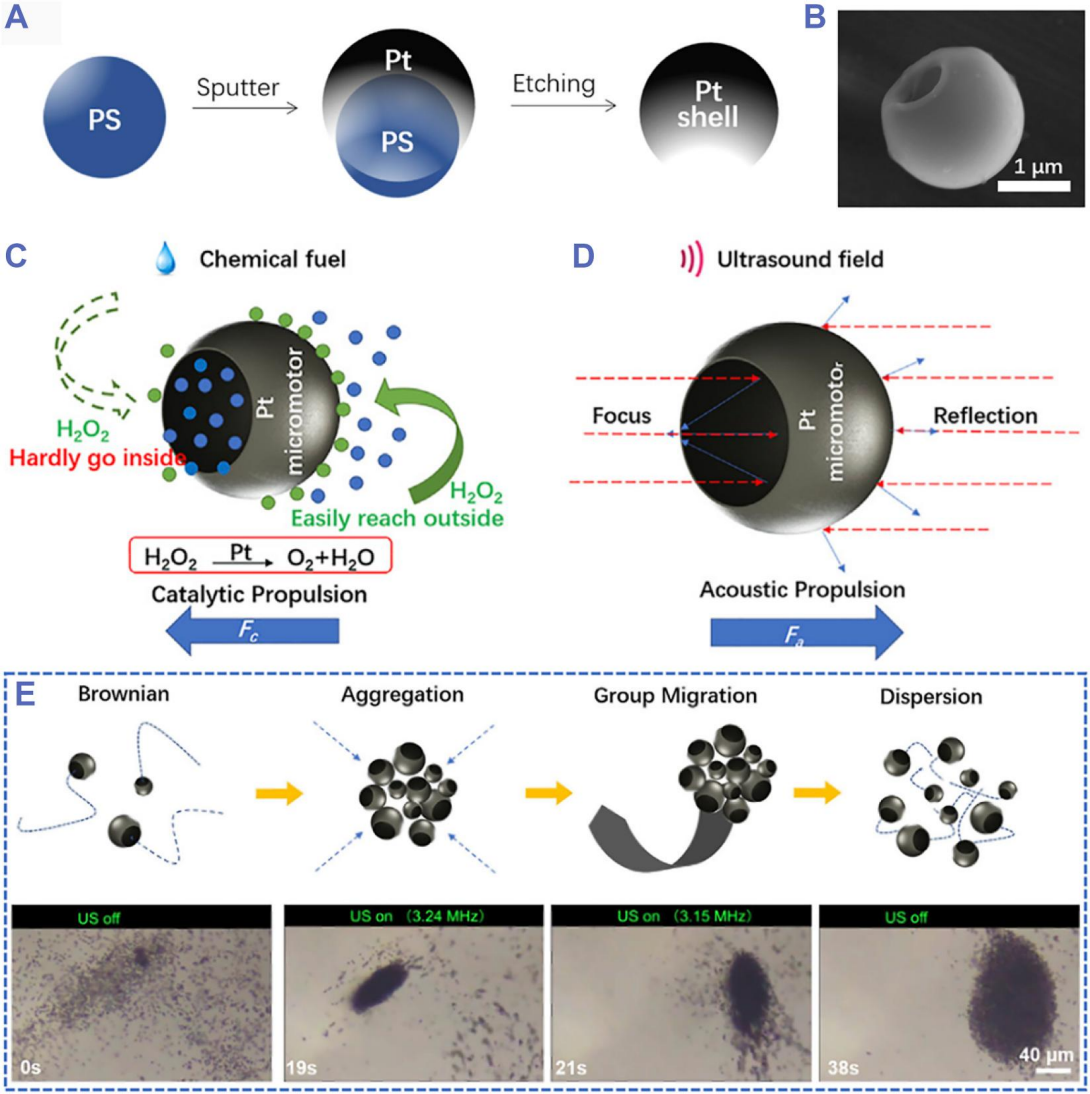
Cup‐shaped platinum (Pt) micromotors with dual‐model propulsion. (A) Schematic illustration of the fabrication process of Pt micromotors. (B) Scanning electron microscope (SEM) image of a Pt micromotor. (C) Scheme of the chemical fuel‐propelling mechanism of Pt micromotors. (D) Scheme of the ultrasound (US)‐actuating mechanism of Pt micromotors. (E) Schemes and real‐time images of the group motion behavior of Pt micromotors under the excitation of US. *Source*: Reproduced under terms of the CC‐BY license.^48^ Copyright 2022, The Authors, published by Frontiers Media S.A.

In addition to the widely employed AuNWs, which inherently have asymmetric structures, researchers have also been working on creating structured objects using advance technologies.^74^, ^75^, ^76^, ^77^, ^78^, ^79^ Taking Li's work for instance, they fabricated a platinum (Pt) micromotor with artificial concave and convex structures through a sphere‐template method (Figure 3A).^48^ Interestingly, the Pt micromotor can be propelled with or without US excitation (Figure 3B). When being exposed to the environment with hydrogen peroxide, Pt can catalyze the fuel to generate oxygen bubbles, actuating the Pt micromotor toward the concave side (Figure 3C); whereas when the Pt is exposed to the environment without hydrogen peroxide while with the presence of US, the Pt micromotor can move toward the convex side (Figure 3D). Moreover, the Pt micromotors showed group motion behavior when excited by the US with different frequencies (Figure 3E).

**FIGURE 4:**
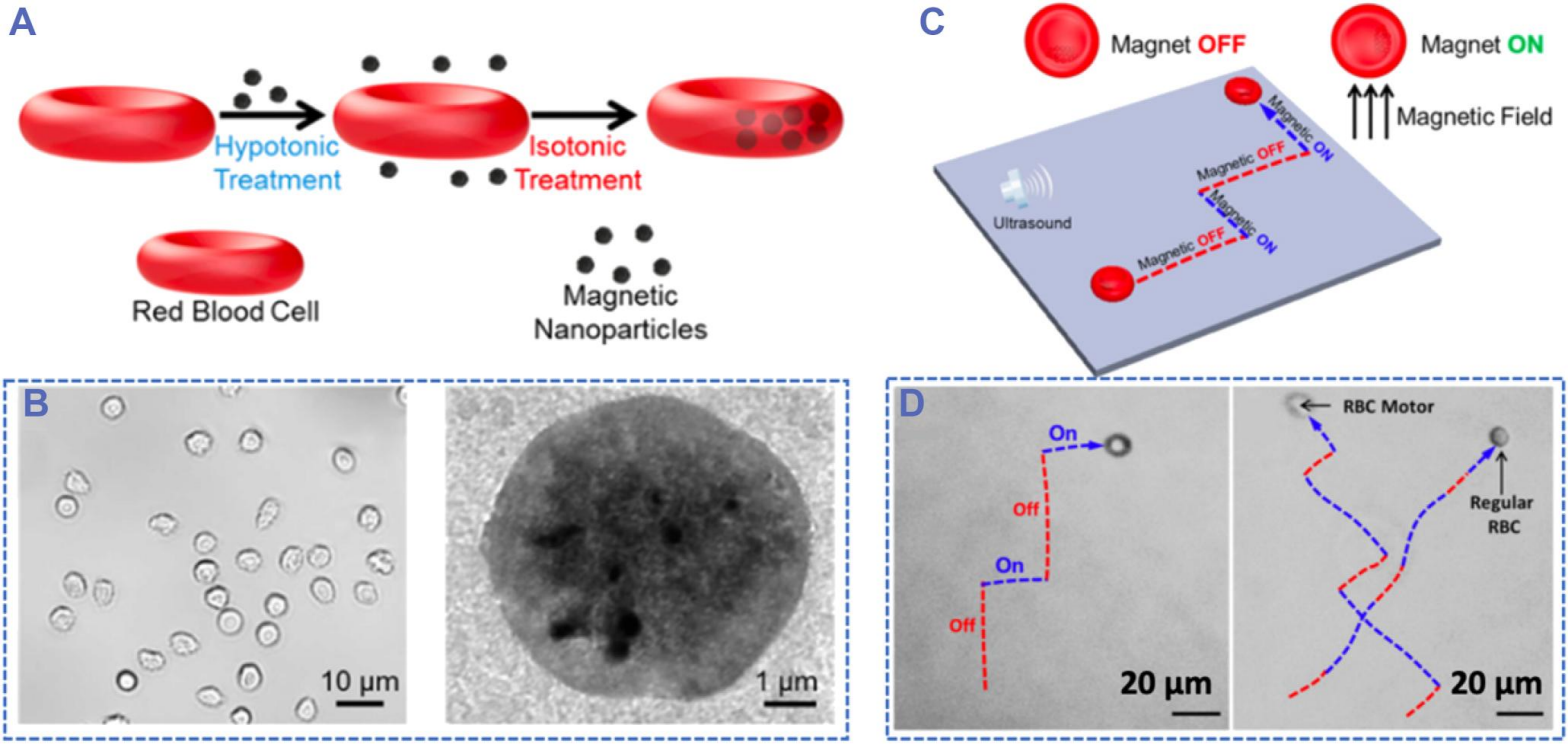
Magnetic nanoparticles loaded red blood cells (RBC) propelled by the ultrasound (US) and navigated by magnetic fields. (A) Schematic illustration of the manufacturing process of RBC robots. (B) Optical image and transmission electron microscopy (TEM) image of the RBC motors. (C) Schematic illustration of magnetic navigation. (D) Real‐time images of the movements of RBC microrobots. *Source*: Reproduced with permission.^46^ Copyright 2014, American Chemical Society.

Similar to the mechanism of US‐actuated NMRs with asymmetric shapes, micro‐ and nanoscale objects with asymmetric density distribution are also potential candidates for US‐propelled robots. The inherent inhomogeneous geometry of the MNRs leads to the acoustic pressure gradient, contributing to the propulsion of MNRs. In addition to distinctive shapes and artful density designs, Wu and co‐workers applied RBCs to load iron oxide nanoparticles to realize asymmetric density distribution as well as prolonged biological circulation and enhanced biosafety (Figure 4A).^46^ By applying the hypotonic dilution encapsulation approach, the magnetic nanoparticles can be efficiently loaded into the RBC (Figure 4B). Interestingly, the iron oxide nanoparticles can respond to magnetic fields, bringing about the US and magnetic dual controllability of the RBC microrobots (Figure 4C,D). These RBC robots were also demonstrated to steer on‐demand in various biological fluids, such as cell culture medium and whole blood.

**FIGURE 5:**
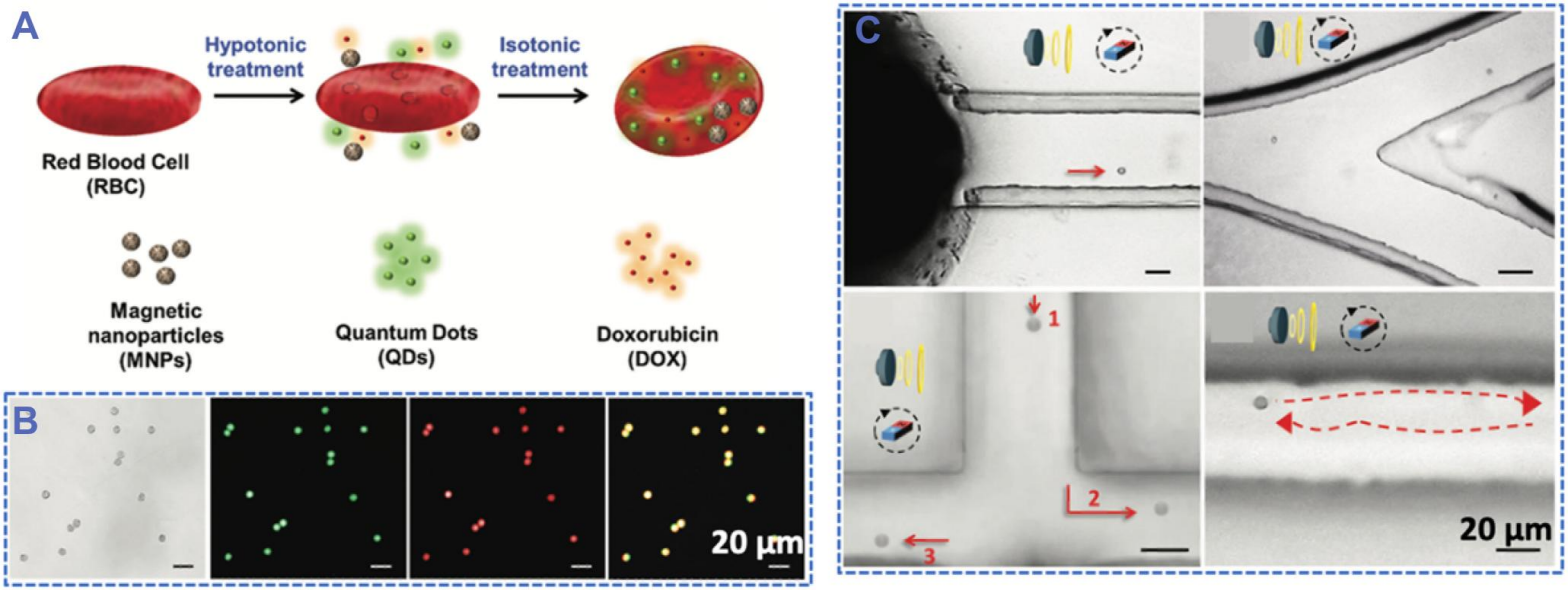
Ultrasound (US)‐propelled multiple agents‐loaded red blood cells (RBC) microrobots. (A) Scheme of the multi‐drug loading process of the RBC microrobots. (B) Bright field and fluorescent images of the RBCs loaded with magnetic nanoparticles, quantum dots, and doxorubicin. (C) Controllable movements of the US and magnetic field dual navigated RBC microrobots. *Source*: Reproduced with permission.^47^ Copyright 2015, The Royal Society of Chemistry.

To further explore the biomedical practicability of RBC‐based microrobots, Wu's group continued to load RBCs with multiple agents (Figure 5A).^47^ In addition to the magnetic‐responsive iron oxide nanoparticles, the RBC microrobots also carried with imaging agents and chemotherapeutics to realize the diagnostic and therapeutic purposed in one single regime. By detecting the green fluorescence emitted from CdTe quantum dots and the red fluorescence emitted from doxorubicin, the successful multi‐cargo upload of the RBC microrobots was demonstrated (Figure 5B). Tested results showed the controllable and efficient movement of the multi‐drug loaded RBC microrobot under the combinational navigation of US and magnetic field (Figure 5C). Works reported by Wu's group vividly illustrated the RBC‐based US‐propelled microrobots feature bright potential in navigated drug delivery and multi‐purpose biomedical applications.

## 4 BUBBLE OSCILLATION PROPULSION

**FIGURE 6:**
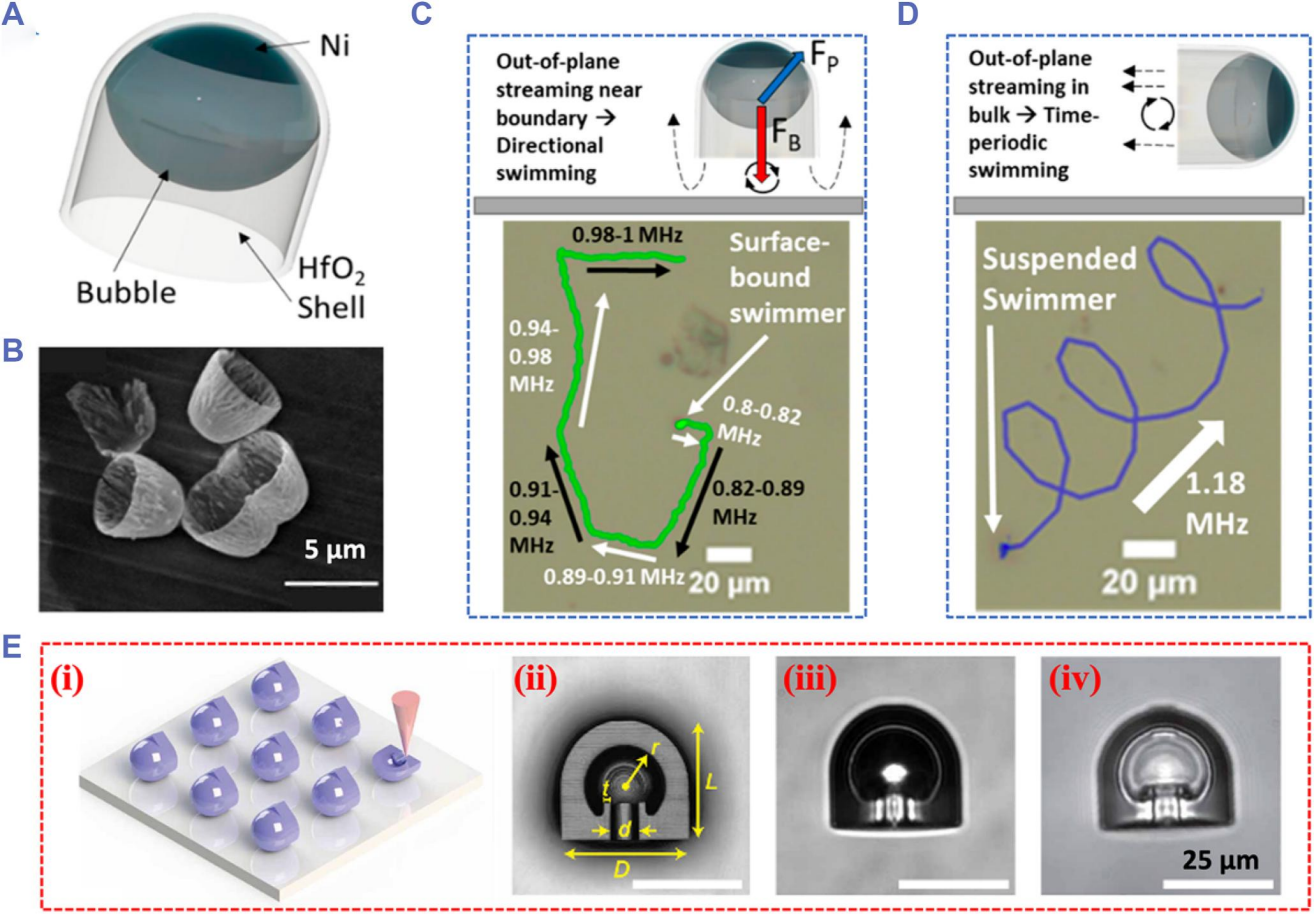
Cup‐shaped microrobots propelled by ultrasound (US)‐oscillated bubbles. (A) Schematic illustration of the cup‐shaped micro‐swimmer. (B) Scanning electron microscope (SEM) image of the micro‐swimmers. (C) Scheme and trajectory of the micro‐swimmer propelled by tuning US frequency. (D) Scheme and trajectory of the micro‐swimmer moving in 3D space under excitation of US with frequency of 1.18 MHz. *Source*: Reproduced with permission.^41^ Copyright 2020, American Chemical Society. (E) (i) Schematic illustration of the fabrication process of the cup‐shaped microrobots. (ii) The cross‐section image of a microrobot. D represents 30 μm, *d* represents 6 μm, *L* represents 27 μm, *r* represents 9 μm, and t represents 2 μm. (iii) Bright field microscope image of a microrobot in PBS. (iv) Bright field microscope image of a microrobot in low surface tension liquid. *Source*: Reproduced under terms of the CC‐BY license.^43^ Copyright 2022, The Authors, published by American Association for the Advancement of Science.

The acoustic streaming actuating MNRs based on asymmetric density or shape have shown outstanding dynamic locomotion and tunability. However, the controllability of these MNRs at the acoustic pressure nodes remains a limitation in practical applications. By contrast, microbubble resonance can serve as an effective and sensitive approach to propel MNRs.^80^, ^81^, ^82^ Generally, bubbles can be formed inside the MNRs upon being quickly immersed into the liquid, by trapping the air inside the cavity initially. US of a specific frequency and power will resonate with bubbles of the corresponding size, and then cause the oscillation of bubbles. The oscillation is transmitted to the liquid through the air–liquid interface, and the reverse propulsion force is generated to render the locomotion of MNRs. To trap bubbles steadily and realize vibration effectively, efforts have been focused on the design of MNRs with numerous shapes, such as tubular, cup‐shaped, bullet‐shaped, and so on. McNeill et al. proposed a series of cup‐shaped micro‐swimmers with various dimensions and scale‐dependent US‐propelled motions via the non‐photolithographic method (Figure 6A,B).^41^ Under the excitation of US with different parameters, these cup‐shaped micro‐swimmers showed different bubble oscillation modes and regulated motion modes that can be dynamically switched between 2D and 3D (Figure 6C,D). Furthermore, Aghakhani's group designed a microrobot with a double reentrant edge and a hollow body to enhance the liquid repellency at the air–liquid interface, contributing to the improved lifetime and stability of the trapped microbubble in different biological fluids (Figure 6E).^43^ They also elaborated the dynamic motion performance of their microrobots in both Newtonian and non‐Newtonian liquids, showing broader potential in practical applications.

**FIGURE 7:**
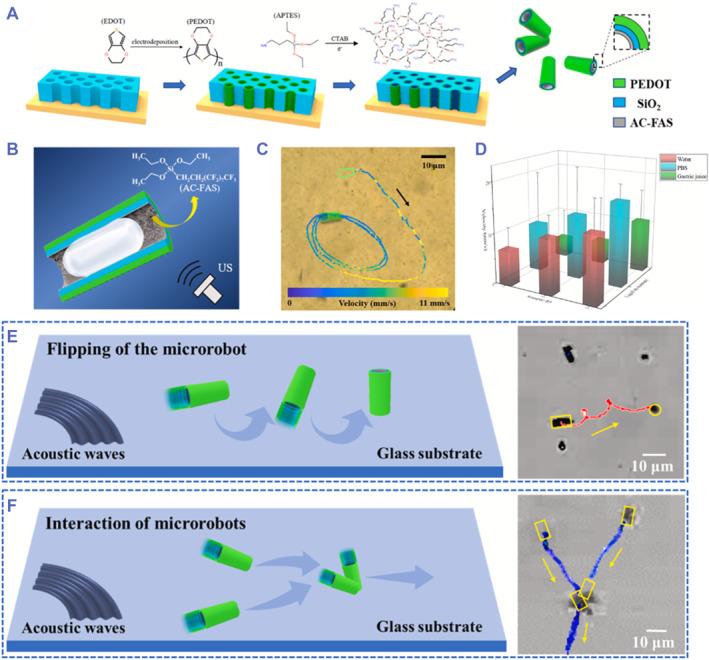
Tubular microrobots with superfast locomotion velocity. (A) Schematic diagram of the high throughput production of tubular microrobots. (B) Scheme of the bubble propulsion mechanism. (C) The trajectory of the EDOT‐SiO_2_ micromotor actuated by ultrasound (US) with frequency of 4.6 MHz and voltage of 15 V. (D) The locomotion velocities of EDOT‐SiO_2_ micromotors in water (red), PBS (blue), and artificial gastric juice (green). (E) Scheme and trajectory of the vertical alignment performance of an EDOT‐SiO_2_ micromotor. (F) Scheme and trajectory of two EDOT‐SiO_2_ micromotors. *Source*: Reproduced with permission.^40^ Copyright 2022, Elsevier.

The hollow tubular shape with two openings or one opening is also of significance in the generation of bubbles. Lu's group developed a tubular US‐propelled microrobot with merits of superfast velocity and high throughput synthesis (Figure 7A).^40^ Like other tubular robots, Lu's robot was able to wrap a bubble inside after immersing into the liquid environment (Figure 7B). Interestingly, Lu et al. employed the electrochemical deposition method to achieve mass and cost‐effective production of the micromotors, compromising the limitations of low yield of the former reported fine‐structured micro‐ and nano‐robots. Based on a poly(3, 4‐ethyl‐enedioxythiophene) (PEDOT) skeleton, silicon dioxide (SiO_2_) was further deposited onto the PEDOT to enhance the mechanical strength of the robot (PEDOT‐SiO_2_ micromotor). Then, the hydrophobic (heptadecafluoro‐1,1,2,2‐tetradecyl) trimethoxy silane (AC‐FAS) was added to the inner layer to capture bubbles. As depicted in the moving trajectories, under US triggering, the PEDOT‐SiO_2_ micromotor can speed up to 11 mm/s within 30 ms, with a velocity which was equivalent to the length of 1100 PEDOT‐SiO_2_ micromotor per second (Figure 7C). Moreover, tested results demonstrated that the PEDOT‐SiO_2_ micromotors also can move superfast both in phosphate‐buffered saline (PBS) (pH 7.4) and artificial gastric juice (pH 1.4) (Figure 7D). Interestingly, it was further observed that the PEDOT‐SiO_2_ micromotors can achieve vertical alignment under US excitation (Figure 7E). Moreover, when two independent PEDOT‐SiO_2_ micromotors get propulsion in the same space, the two micromotors can attract each other, form a V‐shape, and finally move together (Figure 7F). Compared with other US‐propelled micromotors, the PEDOT‐SiO_2_ micromotor featured advantages in high productivity and outstanding motion velocity in numerous biological fluids, showing remarkable potentials in biomedical applications.

**FIGURE 8:**
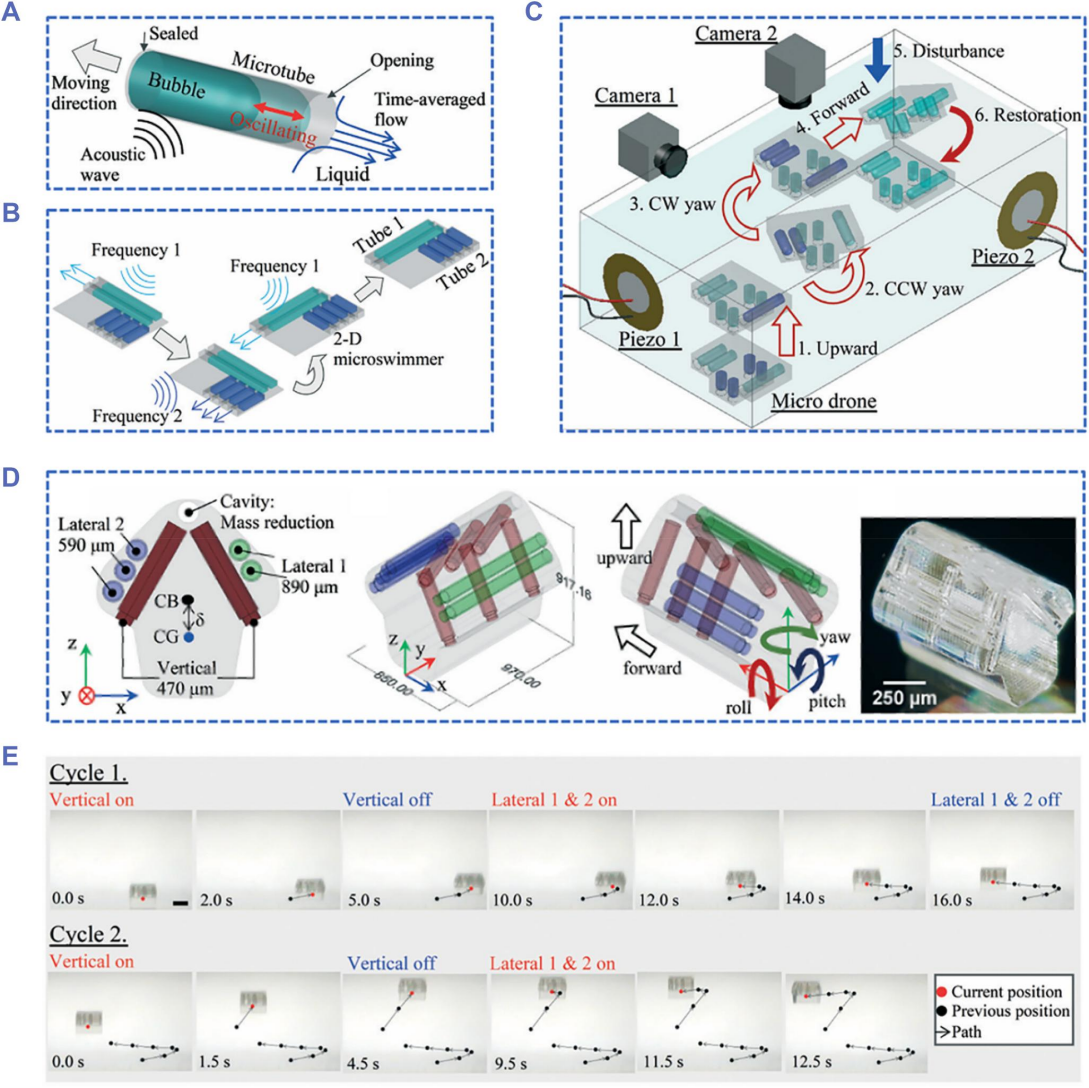
3D printed microdrone propelled by bubble oscillation. (A–C) Schemes of the 1D (A), 2D (B), and 3D (C) movements of single microtube and assemble microtubes propelled by ultrasound (US). (D) Schematic diagram of the microtubes assembled in 3D space and the digital image of a 3D microdrone. (E) The consecutive US actuation and the on‐demand movements of a microdrone. *Source*: Reproduced with permission.^42^ Copyright 2021, The Royal Society of Chemistry.

The microbubbles trapped in the tubes show outstanding US‐sensitivity and effective US propulsion. The speed and direction of the US‐driven motion depend on the size of the bubble and the orientation of the tube opening. According to the length of the one‐end opening tubes, the trapped cylindrica bubbles have different resonance frequencies, at which the bubbles would generate maximum actuation force. Therefore, Liu's group assembled tubes of different lengths in different directions on one device via 3D printing, and then further balanced the mass and shape of the device to obtain a microdrone that can move in a controllable direction at different US frequencies.^42^ In addition to the back‐and‐forth March of the one microtube on 1D plane (Figure 8A), and the actuated motion on x–y plane of the microtubules assembled on 2D plane (Figure 8B), the 3D microdrone with microtubes assembled in multiple directions can realize the lift in the *z*‐direction (Figure 8C), achieving movements in three dimensions. Since bubbles with different lengths can only respond to US with specific frequencies, bubbles of different orientations can be excited by one or more US waves alone or simultaneously, obtaining the forward, upward, clockwise, and counterclockwise actuations of the 3D microdrone (Figure 8D). By consecutively and jointly applying the US with different frequencies, the on‐demand movements of the 3D microdrone in 3D space can be observed (Figure 8E). This flexible and controllable US‐propelled microrobot holds a bright future in biosensing, chemical detection, cargo navigation, or even microsurgery.

## 5 FUTURE PERSPECTIVES

In recent decades, the rapid development of micro‐ and nano‐manufacturing technology has contributed to the advancement of autonomous robots.^11^, ^15^, ^16^, ^39^, ^83^, ^84^, ^85^, ^86^, ^87^, ^88^, ^89^, ^90^, ^91^, ^92^, ^93^, ^94^, ^95^, ^96^, ^97^ In this mini review, we addressed several representative MNRs that can be sensitively actuated by the US. Attributing to the excellent biocompatibility, highly efficient penetrability, and low energy attenuation of US, the US‐propelled MNRs have broader biomedical applications compared with other external energy fields. Typically, micro‐ and nano‐scale objects with asymmetric shape and density can be propelled by the US‐caused unevenly distributed acoustic pressure. In addition to the abovementioned AuNWs, numerous MNRs with asymmetric geometry have been presented and showed myriad applications, such as twisted star‐shaped Au microplates,^49^ composite nickel and gold object with a polypyrrole flagellum,^51^ Janus microparticle with a silica core and a platinum cap,^45^ etc. Efforts have been focused to enhance the biocompatibility of these composite metallic materials and have achieved some progress. The biocompatible MNRs can break through the biological barriers and aggregate effectively in targeted cells. However, the biodegradability and biological circulation of these nanomaterials remain challenges in practical applications, advancements on the front of which are still anticipated.

The MNRs actuated by bubble oscillation have excellent maneuverability and on‐demand controllability. Ascribing to the tremendous development of 3D printing, which allows fabrication of nano‐precision objects,^98^, ^99^, ^100^, ^101^, ^102^, ^103^, ^104^ more and more sophisticated MNRs have merged. Whereas, the lifetime and stability of the trapped bubbles play a critical role in the prolonged propulsion of MNRs. Some studies applied poor water‐soluble gases to pretreat the MNRs, and some studies coated the MNRs with hydrophobic polymers. In order to make US‐actuated MNRs into practical applications, large‐scale and efficient production of MNRs is also an important part. Typically, Lu's study showed us a novel high‐throughput MNRs manufacture method based on electrochemical deposition technology, enlightening us the exploration of more agile and tunable means to fabricate MNRs.^40^ Besides, different MNRs are responsive to US with different frequencies and intensities, the optimal US parameters require further exploration. The development of US‐propelled MNRs reflects the broad and potential application of autonomous robots from a side. Therefore, intelligent MNRs with multifaceted and multidisciplinary practicability are highly anticipated.

## AUTHOR CONTRIBUTIONS

Yuanjin Zhao conceived the project. Danqing Huang wrote the manuscript and arranged the figures. Lijun Cai and Ning Li revised the manuscript.

## CONFLICT OF INTEREST STATEMENT

The authors declare no conflict of interest.
